# Association of Body Weight Variability With Progression of Coronary Artery Calcification in Patients With Predialysis Chronic Kidney Disease

**DOI:** 10.3389/fcvm.2021.794957

**Published:** 2022-01-26

**Authors:** Sang Heon Suh, Tae Ryom Oh, Hong Sang Choi, Chang Seong Kim, Eun Hui Bae, Kook-Hwan Oh, Kyu-Beck Lee, Seung Hyeok Han, Suah Sung, Seong Kwon Ma, Soo Wan Kim

**Affiliations:** ^1^Department of Internal Medicine, Chonnam National University Medical School and Chonnam National University Hospital, Gwangju, South Korea; ^2^Department of Internal Medicine, Seoul National University Hospital, Seoul, South Korea; ^3^Department of Internal Medicine, Kangbuk Samsung Hospital, Sungkyunkwan University School of Medicine, Seoul, South Korea; ^4^Department of Internal Medicine, College of Medicine, Institute of Kidney Disease Research, Yonsei University, Seoul, South Korea; ^5^Department of Internal Medicine, Eulji Medical Center, Eulji University, Seoul, South Korea

**Keywords:** body weigh variability, cardiovascular disease, chronic kidney disease, coronary artery calcification, cardiovascular event

## Abstract

**Background:**

We investigated whether high body weight variability (BWV) is associated with a higher prevalence of coronary artery calcification (CAC) or more rapid progression of CAC in patients with predialysis chronic kidney disease (CKD).

**Methods:**

A total of 1,162 subjects from a nationwide prospective cohort of predialysis CKD were analyzed. The subjects were divided into the tertile (T1, T2, and T3) by BWV. CAC was assessed at the baseline and a 4-year follow-up by CT scan. Rapid progression of coronary artery calcification was defined as an increase in coronary artery calcium score (CACS) more than 200 Agatston units during a 4-year follow-up.

**Results:**

One-way ANOVA revealed that CACS change during the follow-up period is significantly higher in the subjects with high BWV, although CACS at the baseline and 4-year follow-up was not different among the tertile groups by BWV. Logistic regression analysis revealed that compared to low BWV (T1), both moderate (T2, adjusted odds ratio (OR) 2.118, 95% CI 1.075–4.175) and high (T3, adjusted OR 2.602, 95% CI 1.304–5.191) BWV was associated with significantly increased risk of rapid progression of CAC. Importantly, the association between BWV and progression of CAC remained robust even among the subjects without significant BW gain or loss during follow-up periods (T2, adjusted OR 2.007, 95% CI 1.011–3.984; T3, adjusted OR 2.054, 95% CI 1.003–4.207).

**Conclusion:**

High BWV is independently associated with rapid progression of CAC in patients with predialysis CKD.

## Introduction

Patients with chronic kidney disease (CKD) are likely to experience body weight (BW) fluctuation. Accumulation of excess extracellular fluid in patients with CKD is associated with BW gain, leading to accelerated coronary artery calcification (1), cardiovascular (CV) remodeling (2), and increase the risk of adverse renal outcome (3), and all-cause mortality (4). Conversely, malnutrition-inflammation, which is prevalent even before the commencement of renal replacement therapy (5, 6), is associated with BW loss and is ultimately associated with increased mortality (7). Diuretic use further increases the odd for BW fluctuation in patients with CKD (8). As all these conditions are likely to take place concurrently, it is expected that bodyweight variability (BWV), rather than persistent loss or gain of BW, maybe of high clinical significance, which has not been established especially in patients with predialysis CKD.

Body weight variability is an emerging surrogate of clinical outcomes. BWV is associated with a higher risk of incident diabetes mellitus (DM) (9, 10), and is also associated with adverse CV outcomes (11) and all-cause mortality (9, 12, 13) in the general population. The prognostic impact of BWV has been also validated in more specific clinical contexts, which is well illustrated in patients with DM (14–16) and coronary artery disease (CAD) (17). Moreover, it is noticeable that the association of high BWV with adverse CV outcomes is independent of traditional CV risk factors (15, 17), suggesting a potential role of BWV in the prediction of outcomes in patients with CKD. In this regard, we recently reported that high BWV is associated with adverse CV outcomes in patients with predialysis CKD (18). Despite the robust association between BWV and CV outcomes in this population, the precise mechanism of how BWV is linked to CV events was not clearly evaluated yet.

The high prevalence and prognostic role of coronary artery calcification (CAC) in patients with CKD have been investigated in several studies (19). Therefore, we here hypothesized that high BWV would be associated with a higher prevalence of CAC or more rapid progression of CAC lesions in patients with predialysis CKD, as CKD is an independent risk factor for the development of CAD (20), and CAD is the leading cause of mortality in patients with CKD (21). We analyzed the coronary artery calcium score (CACS) at the baseline and 4-year follow-up by the degree of visit-to-visit BWV in patients with predialysis CKD. To examine the clinical significance of BWV that is distinctive from longitudinal BW change, we also investigated the association of BWV with CACS progression in patients without significant BW gain or loss during follow-up periods.

## Methods

### Study Designs and Participants

**Figure 1:**
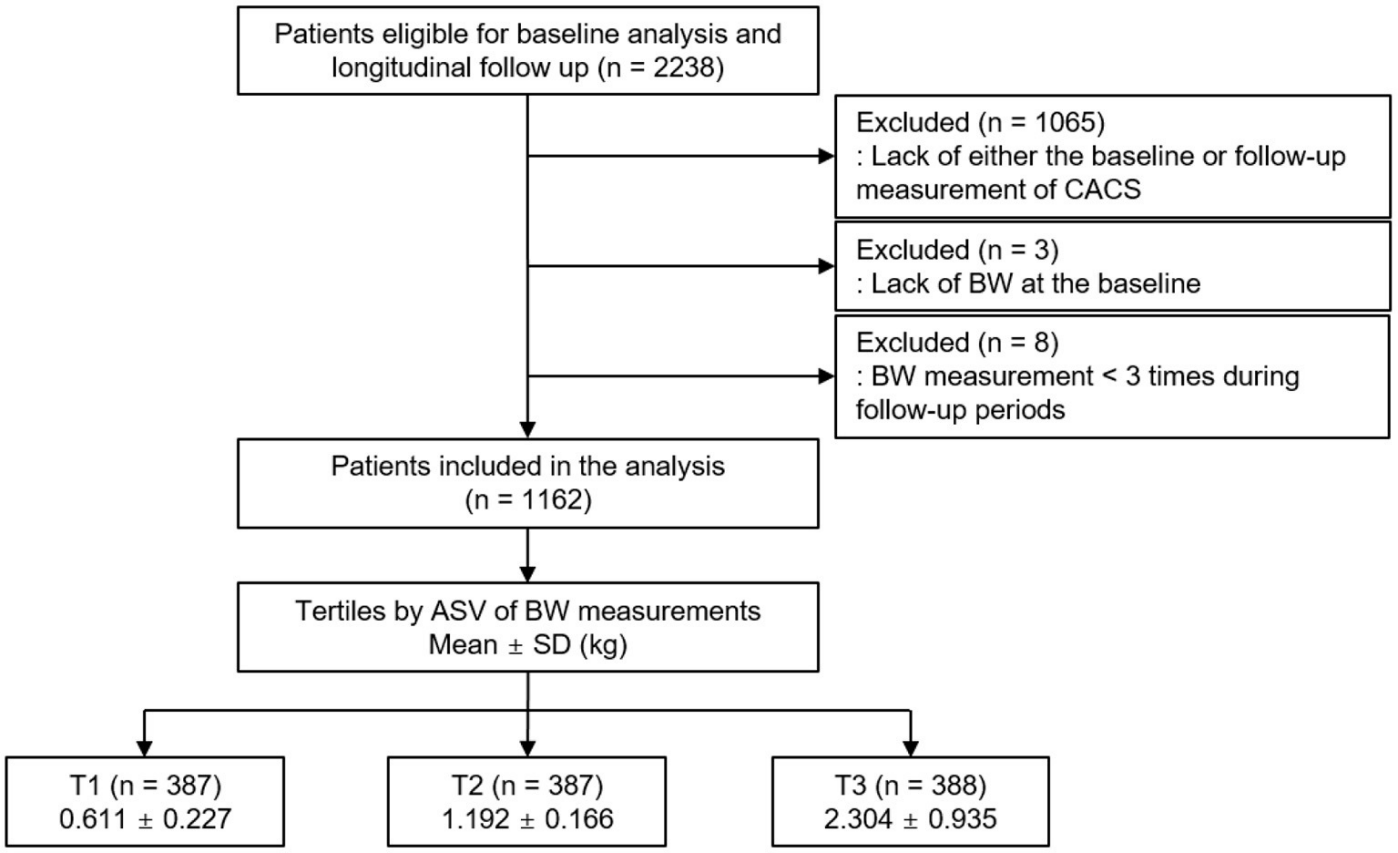
Flow diagram of the study participants. ASV, average successive variability; BW, body weight; CACS, coronary artery calcium score; T1, 1st tertile; T2, 2nd tertile; T3, 3rd tertile.

The Korean Cohort Study for Outcomes in Patients With Chronic Kidney Disease (KNOW-CKD) is a nationwide prospective cohort study involving nine tertiary-care general hospitals in Korea (NCT01630486 at http://www.clinicaltrials.gov) (22). Korean patients with CKD from stage 1 to predialysis stage 5, who voluntarily provided informed consent were enrolled, according to the previously described inclusion and exclusion criteria (22). This study was conducted following the principles of the Declaration of Helsinki, and the study protocol was approved by the institutional review boards of participating centers, including at the Seoul National University Hospital, the Yonsei University Severance Hospital, the Kangbuk Samsung Medical Center, the Seoul St. Mary's Hospital, the Gil Hospital, the Eulji General Hospital, the Chonnam National University Hospital, and the Busan Paik Hospital. A total of 2,238 subjects were longitudinally followed up (Figure 1). After excluding those lacking either the baseline or follow-up measurement of CACS, those lacking the baseline BW measurement, and those with the number of BW measurements during follow-up periods less than three times, 1,162 subjects were finally included for further analyses. The median follow-up duration was 6.940 years.

### Data Collection

Demographic data, including age, gender, smoking history, medications (angiotensin-converting enzyme inhibitors and angiotensin receptor blockers (ACEi/ARBs), diuretics, number of antihypertensive drugs, and statins), and comorbid conditions, were collected at the time of screening. Anthropometric indices [height, weight, and systolic and diastolic blood pressures (SBP and DBP)] were also measured. Body mass index (BMI) was calculated as weight/height^2^ (kg/m^2^). Laboratory data included hemoglobin, creatinine, albumin, glucose, triglyceride, total cholesterol, low-density lipoprotein cholesterol, high-density lipoprotein cholesterol (HDL-C), 25-hydroxyvitamin D (25(OH) vitamin D), and high sensitive C-reactive protein (hs-CRP). Serum creatinine was measured by an isotope dilution mass spectrometry–traceable method, and estimated glomerular filtration rate was calculated by Chronic Kidney Disease Epidemiology Collaboration equation (23). CKD stages were determined by the Kidney Disease Improving Global Outcomes guidelines (24). Urine albumin-to-creatinine ratio (ACR) was measured in random, preferably first-voided, spot urine samples.

### Determination of BWV

Body weight was measured at 0, 6, and 12 months and then yearly thereafter up to 8 years. The median number of BW measurements was seven times. Intra-individual visit-to-visit BWV is determined by average successive variability, defined as the average absolute difference between successive values (9, 14, 17, 25). The subjects were divided into the tertile by BWV, in which the 1st (T1), 2nd (T2), and 3rd (T3) tertiles were defined as low, moderate, and high BWV, respectively (Figure 1).

### Estimation of the Rate of Longitudinal BW Change During Follow-Up Periods

The rate of longitudinal BW change for each individual was estimated using a regression model and expressed as the slope (%/year) (26). BW change less than 2.5%/year was defined as non-significant BW gain or loss (26) (Supplementary Table S1).

### Measurement of CACS and Study Outcome

Electrocardiography-gated coronary multidetector CT scans were checked following the standard protocol of each center at the baseline and year four follow-up visit. The CACS score was determined using the Agatston unit (AU) on a digital radiologic workstation (27). Rapid progression of coronary artery calcification was defined as an increase in coronary artery calcium score (CACS) more than 200 AU during a 4-year follow-up (28).

### Statistical Analysis

Continuous variables were expressed as mean ± SD or median [interquartile range]. Categorical variables were expressed as the number of participants and percentage. For descriptive analyses, the Student's *t*-test or one-way ANOVA and the chi-squared test were used for continuous and categorical variables, respectively. A binary logistic regression model was analyzed to address an independent association between BWV and rapid progression of CAC, multivariate logistic regression models were analyzed. The models were adjusted for age, gender, Charlson comorbidity index, smoking history, BMI, SBP, DBP, medications (ACEi/ARBs, diuretics, total number of antihypertensive drugs, and statins), hemoglobin, albumin, HDL-C, fasting glucose, hs-CRP, 25(OH) vitamin D levels, eGFR, spot urine ACR, and baseline CACS. The results of binary logistic regression analysis were presented as odds ratios (ORs) and 95% CI. Restricted cubic splines were used to visualize the association between serum adiponectin as a continuous variable and the OR for fatal and nonfatal CV events or all-cause mortality. For sensitivity analysis, first, multivariate linear regression analysis was performed to test the linear association of BWV and CACS change during follow-up periods. Second, those with CACS <10 AU at the baseline were excluded for binary logistic regression analysis, as the progression of CAC in the subjects was relatively rare (29). Third, as we assumed that the BWV may be exaggerated in advanced CKD, those with CKD stages 4 and 5 at the baseline were excluded for binary logistic regression analysis. Finally, to examine the clinical significance of BWV that is distinctive from longitudinal BW change, those without significant BW gain or loss during follow-up periods were excluded for binary logistic regression analysis. The results of multivariate linear regression analysis were presented as β coefficient and 95% CI. Two-sided *P* < 0.05 were considered statistically significant. Statistical analysis was performed using SPSS for Windows version 22.0 (IBM Corp., Armonk, NY, USA).

## Results

### Baseline Characteristics

The baseline characteristics of study participants in the tertile by BWV are described in Table 1. The mean age, the frequency of male gender, and the burden of comorbid conditions significantly increased as BWV increased. Accordingly, the frequency of the subjects with a history of DM and the subjects on medication of no less than three antihypertensive drugs increased as BWV increased. All the other demographic data and medical history did not differ significantly across the groups. BMI also increased as BWV increased. SBP and DBP were not significantly different among the groups. Serum albumin, triglyceride, and fasting glucose levels increased as BWV increased. In contrast, HDL-C and 25(OH) vitamin D levels decreased as BWV increased. The other laboratory findings, such as spot urine ACR and eGFR, were not significantly different among the tertile groups.

**Table 1:** Baseline characteristics of study participants in the tertile by BWV.

	**BWV**			
	**T1**	**T2**	**T3**	***P*** **-value**
Follow-up duration (year)	6.466 ± 1.450	6.569 ± 1.413	6.466 ± 1.450	0.594
CACS (AU)				0.804
0	216 (55.8)	198 (51.2)	204 (52.6)	
0 <, ≤ 400	178 (38.2)	157 (40.6)	155 (39.9)	
400 <, ≤ 1000	14 (3.6)	22 (5.7)	20 (5.2)	
1,000 <	9 (2.3)	10 (2.6)	9 (2.3)	
Age (year)	54.065 ± 10.334	53.390 ± 11.171	50.353 ± 13.246	<0.001
Male	208 (53.7)	229 (59.2)	253 (65.2)	0.005
Charlson comorbidity index				0.007
0–3	340 (87.9)	312 (80.6)	304 (78.4)	
4–5	44 (11.4)	73 (18.9)	81 (20.9)	
6–7	3 (0.8)	2 (0.5)	3 (0.8)	
DM	72 (18.6)	107 (27.6)	121 (31.2)	0.001
CAD	2 (0.5)	9 (2.3)	10 (2.6)	0.162
Arrhythmia	8 (2.1)	3 (0.8)	4 (1.0)	0.426
Medication				
ACEi/ARBs	336 (86.8)	344 (88.9)	327 (84.3)	0.167
Diuretics	91 (23.5)	91 (23.5)	108 (27.8)	0.276
Number of anti-HTN drugs ≥ 3	78 (20.2)	81 (20.9)	111 (28.6)	0.009
Statins	204 (52.7)	192 (49.6)	186 (47.9)	0.403
BMI (kg/m^2^)	23.788 ± 2.2924	24.363 ± 3.058	25.616 ± 3.746	<0.001
SBP (mmHg)	125.295 ± 14.014	125.645 ± 14.326	126.894 ± 15.551	0.279
DBP (mmHg)	77.220 ± 9.984	76.259 ± 10.358	77.090 ± 10.766	0.377
Laboratory findings				
Hemoglobin (g/dl)	13.261 ± 1.664	13.414 ± 1.887	13.408 ± 1.946	0.429
Albumin (g/dl)	4.226 ± 0.333	4.273 ± 0.335	4.296 ± 0.363	0.016
Total cholesterol (mg/dl)	174.434 ± 35.000	174.179 ± 36.672	175.917 ± 35.720	0.768
HDL-C (mg/dl)	52.278 ± 15.914	51.237 ± 15.047	48.970 ± 14.017	0.008
LDL-C (mg/dl)	95.937 ± 29.055	96.560 ± 30.792	98.829 ± 30.224	0.375
TG (mg/dl)	142.621 ± 91.443	151.832 ± 91.224	162.992 ± 101.510	0.013
Fasting glucose (mg/dl)	102.632 ± 25.048	109.135 ± 33.699	108.190 ± 31.623	0.006
25(OH) vitamin D	18.835 ± 7.286	18.854 ± 7.712	17.180 ± 6.765	0.001
hsCRP (mg/dl)	0.600 [0.200, 1.400]	0.510 [0.200, 1.400]	0.700 [0.200, 1.900]	0.809
Spot urine ACR (mg/gCr)	245.079 [42.450, 616.708]	217.470 [34.877, 597.054]	241.744 [56.312, 625.118]	0.623
eGFR (ml/min./1.73 m^2^)	57.441 ± 27.405	59.227 ± 28.337	61.375 ± 30.714	0.165
CKD stages				0.167
Stage 1	72 (18.6)	79 (20.4)	100 (25.8)	
Stage 2	96 (24.8)	101 (26.1)	97 (25.0)	
Stage 3a	84 (21.7)	78 (20.2)	63 (16.2)	
Stage 3b	91 (23.5)	88 (22.7)	79 (20.4)	
Stage 4	41 (10.6)	41 (10.6)	44 (11.3)	
Stage 5	3 (0.8)	0 (0.0)	5 (1.3)	

*Values for categorical variables are given as number (percentage); values for continuous variables, as mean ± SD or median (interquartile range)*.

*ACEi, angiotensin-converting enzyme inhibitor; ACR, albumin-to-creatinine ratio; ARB, angiotensin receptor blocker; AU, Agatston unit; BMI, body mass index; BWV, body weight variability; CACS, coronary artery calcium score; CAD, coronary artery disease; CKD, chronic kidney disease; Cr, creatinine; DBP, diastolic blood pressure; DM, diabetes mellitus; eGFR, estimated glomerular filtration rate; HDL-C, high density lipoprotein cholesterol; hs-CRP, high-sensitivity C-reactive protein; HTN, hypertension; LDL-C, low-density lipoprotein cholesterol; SBP, systolic blood pressure; T1, 1st tertile; T2, 2nd tertile; T3, 3rd tertile; TG, triglyceride; 25(OH) vitamin D, 25-hydroxyvitamin D*.

### Association of BWV and Rapid Progression of CAC in Patients With Predialysis CKD

**Figure 2:**
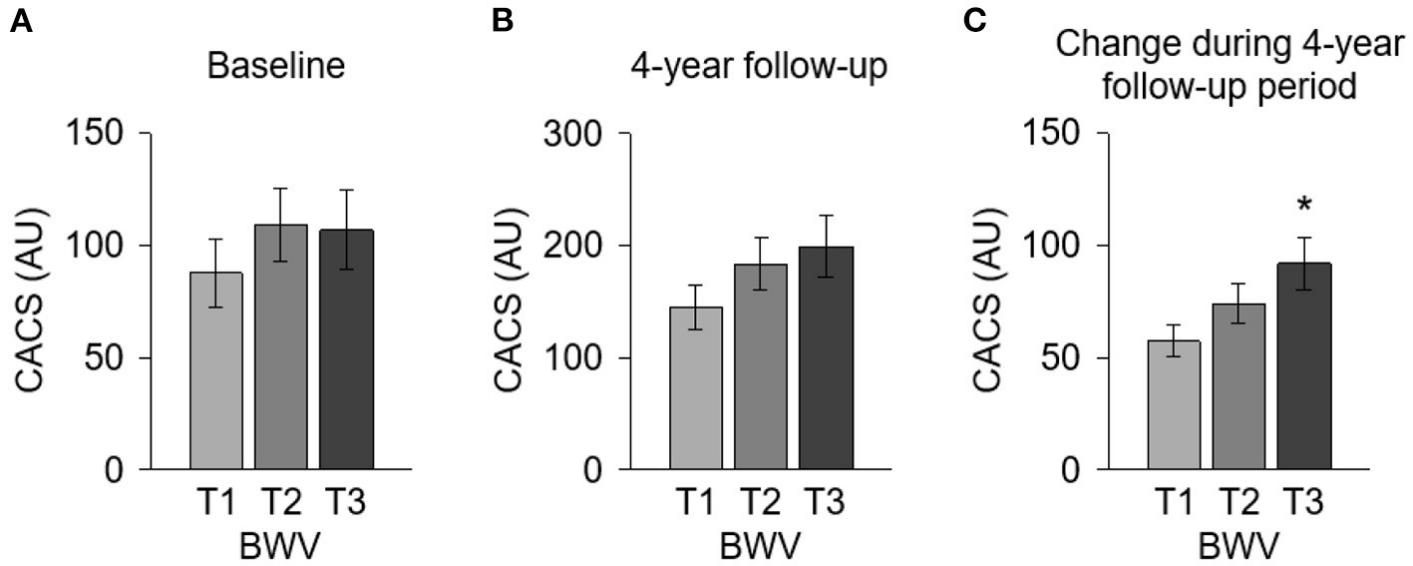
Comparison of the CACS at the baseline and at 4-year follow-up and the CACS change during 4-year follow-up period by BWV. CACS at the baseline **(A)** and at 4-year follow-up **(B)** and the CACS change during the 4-year follow-up period **(C)** were compared by BWV. *P < 0.05 vs. T1 by one-way ANOVA with Scheffe's *post-hoc* test. Error bars indicate SE of means. AU, Agatston unit; BWV, body weight variability; CACS, coronary artery calcium score; T1, 1st tertile; T2, 2nd tertile; T3, 3rd tertile.

**Figure 3:**
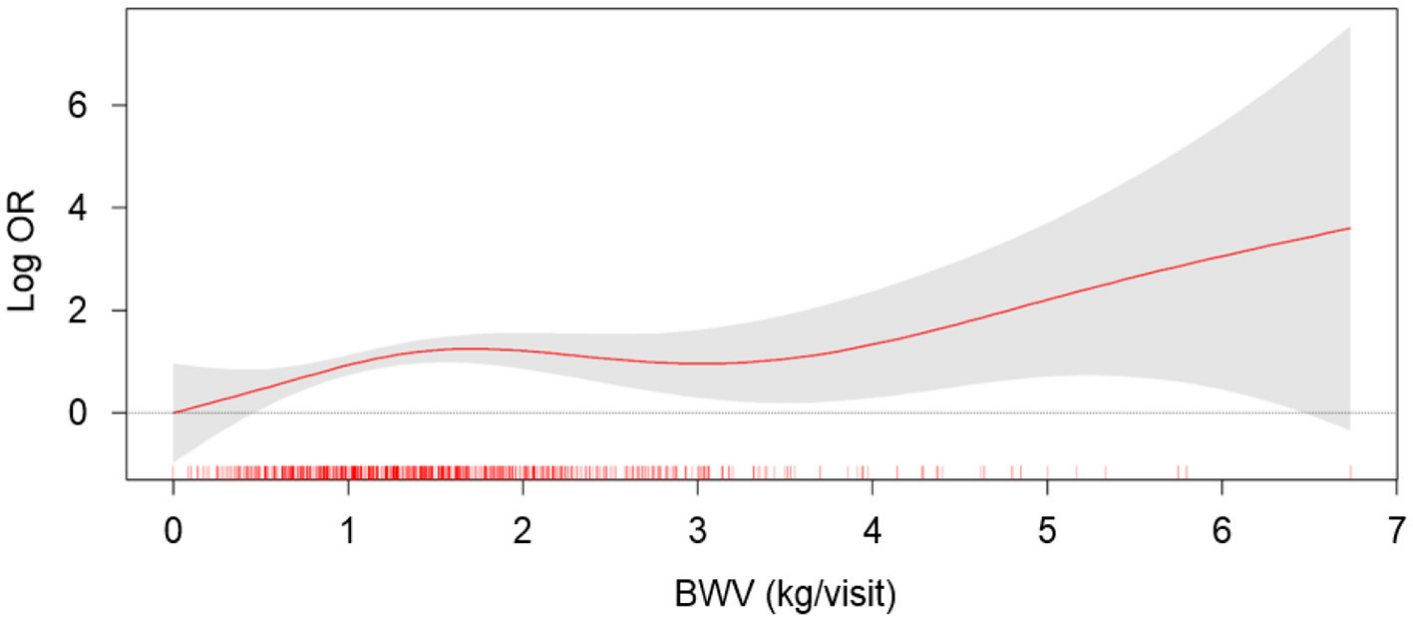
The restricted cubic spline of BWV on the risk of rapid progression of CAC. Adjusted OR of BWV as a continuous variable for the risk of rapid progression of CAC is depicted. The model was adjusted for age, gender, Charlson comorbidity index, smoking history, BMI, SBP, DBP, medication (ACEi/ARBs, diuretics, number of antihypertensive drugs, statins), hemoglobin, albumin, HDL-C, fasting serum glucose, 25(OH) vitamin D, hs-CRP, eGFR, spot urine ACR, and baseline CACS. BWV, body weight variability; OR, odds ratio.

To compare the CACS by BWV, a one-way ANOVA was performed (Figure 2). CACS at the baseline (Figure 2A) was not significantly different among the groups. Although CACS at 4-year follow-up (Figure 2B) was not significantly different among the groups either, CACS at 4-year follow-up gradually increased as BWV increased. Importantly, the change of CACS during the 4-year follow-up period in subjects with high BWV (T2) was significantly larger than that in subjects with low BWV (T1) (Figure 2C). The proportion of the subjects with rapid progression of CAC during the 4-year follow-up period also increased as BWV increased (Supplementary Figure S1). To determine the independent association of BWV with the rapid progression of CAC, a binary logistic regression model was analyzed (Table 2). Compared to low BWV (T1), both moderate (T2, adjusted OR 2.118, 95% CI 1.075–4.175) and high (T3, adjusted OR 2.602, 95% CI 1.304–5.191) BWV was associated with a significantly increased risk of rapid progression of CAC. Restricted cubic spine depicted that adjusted (Figure 2B) OR for rapid progression of CAC is positively correlated with BWV (Figure 3).

**Table 2:** Binary logistic regression of BWV for rapid progression of CAC.

	**Unadjusted**		**Adjusted**	
	**OR (95%CIs)**	***P*** **-value**	**OR (95%CIs)**	***P*** **-value**
BWV, T1	Reference		Reference	
BWV, T2	2.021 (1.194, 3.422)	0.009	2.118 (1.075, 4.175)	0.030
BWV, T3	2.121 (1.259, 3.572)	0.005	2.602 (1.304, 5.191)	0.007

*Models were adjusted for age, gender, Charlson comorbidity index, smoking history, BMI, SBP, DBP, medication (ACEi/ARBs, diuretics, number of antihypertensive drugs, statins), hemoglobin, albumin, HDL-C, fasting serum glucose, 25(OH) vitamin D, hs-CRP, eGFR, spot urine ACR, and baseline CACS*.

*BWV, body weight variability; OR, odds ratio; T1, 1st tertile; T2, 2nd tertile; T3, 3rd tertile*.

### Sensitivity Analysis

To test the linear association of BWV and CACS change during follow-up periods, multivariate linear regression models were analyzed (Supplementary Table S2). The analysis of all subjects revealed that BWV is linearly associated with CACS change during the 4-year follow-up period (adjusted β coefficient 12.098, 95% CI 1.807–22.389). Next, as the progression of CAC is dependent on the baseline CAC status, the subjects with CACS <10 AU at the baseline (*n* = 764) were excluded for binary logistic regression analysis (Supplementary Table S3). Despite the substantial reduction of the number of subjects being analyzed, the analysis revealed a robust association between BWV and rapid progression of CAC (T2, adjusted OR 2.331, 95% CI 1.151–4.718; T3, adjusted OR 2.380, 95% CI 1.148–4.932). In addition, based on the assumption that BWV may be exaggerated in advanced CKD, those with CKD stages 4 and 5 at the baseline (*n* = 134) were excluded for binary logistic regression analysis (Supplementary Table S4). The analysis demonstrated a significant association between BWV and the rapid progression of CAC among the subjects with eGFR ≥ 30 ml/min/1.73 m^2^ (T2, adjusted OR 2.291, 95% CI 1.078–4.867; T3, adjusted OR 3.328, 95% CI 1.529–7.242). Finally, to prove the clinical significance of BWV that is distinctive from longitudinal BW change, those without significant BW gain or loss during follow-up periods (*n* = 69) were excluded for binary logistic regression analysis (Table 3). The analysis reproduced a robust association between BWV and rapid progression of CAC among the subjects without significant BW gain or loss during follow-up periods (T2, adjusted OR 2.007, 95% CI 1.011–3.984; T3, adjusted OR 2.054, 95% CI 1.003–4.207).

**Table 3:** Binary logistic regression of BWV for rapid progression of CAC in subjects with BW maintenance during follow-up periods.

	**Unadjusted**		**Adjusted**	
	**OR (95%CIs)**	***P*** **-value**	**OR (95%CIs)**	***P*** **-value**
BWV, T1	Reference		Reference	
BWV, T2	1.925 (1.130, 3.280)	0.016	2.007 (1.011, 3.984)	0.046
BWV, T3	1.910 (1.110, 3.287)	0.019	2.054 (1.003, 4.207)	0.049

*Models were adjusted for age, gender, Charlson comorbidity index, smoking history, BMI, SBP, DBP, medication (ACEi/ARBs, diuretics, number of antihypertensive drugs, statins), hemoglobin, albumin, HDL-C, fasting serum glucose, 25(OH) vitamin D, hs-CRP, eGFR, spot urine ACR, and baseline CACS*.

*BWV, body weight variability; OR, odds ratio; T1, 1st tertile; T2, 2nd tertile; T3, 3rd tertile*.

### Subgroup Analysis

To figure out whether the association of BWV with the rapid progression of CAC is modified in various clinical contexts, we conducted subgroup analyses. The subgroups were stratified by age (<60 or ≥ 60 years), gender (male or female), Charlson comorbidity index (≤ 3 or ≥ 4), history of DM (without or with), eGFR (≥ 60 or <60 ml/min/1.73 m^2^), spot urine ACR (<300 or ≥ 300 mg/g). Binary logistic analyses revealed that *P* for interactions was > 0.05 for all subgroups (Table 4). Linear regression analysis revealed the association is marginally more significant in those with Charlson comorbidity index ≥ 4 than in those with Charlson comorbidity index ≤ 3 (*P* for interaction = 0.04) (Supplementary Table S2). Age, gender, history of DM, eGFR, and albuminuria did not alter the association between BWV and rapid progression of CAC.

**Table 4:** Binary logistic regression of BWV for rapid progression of CAC in various subgroups.

		**Unadjusted OR (95%CIs)**	***P*** **for interaction**	**Adjusted OR (95%CIs)**	***P*** **for interaction**
Age <60 years	BWV, T1	Reference	0.172	Reference	0.176
	BWV, T2	1.461 (0.663, 3.220)		1.224 (0.330, 4.533)	
	BWV, T3	1.400 (0.642, 3.053)		1.445 (0.347, 6.007)	
Age ≥ 60 years	BWV, T1	Reference		Reference	
	BWV, T2	2.676 (1.283, 5.578)		3.167 (1.237, 8.104)	
	BWV, T3	3.696 (1.767, 7.730)		4.462 (1.729, 11.517)	
Male	BWV, T1	Reference	0.435	Reference	0.719
	BWV, T2	1.674 (0.918, 3.055)		2.193 (0.996, 4.830)	
	BWV, T3	1.590 (0.880, 2.871)		2.395 (1.068, 5.371)	
Female	BWV, T1	Reference		Reference	
	BWV, T2	3.184 (0.991, 10.235)		2.428 (0.293, 20.118)	
	BWV, T3	3.748 (1.163, 12.073)		10.033 (1.088, 92.508)	
Charlson comorbidity index ≤ 3	BWV, T1	Reference	0.858	Reference	0.582
	BWV, T2	0.812 (0.297, 2.221)		1.091 (0.223, 5.343)	
	BWV, T3	0.767 (0.281, 2.097)		1.906 (0.396, 9.163)	
Charlson comorbidity index ≥ 4	BWV, T1	Reference		Reference	
	BWV, T2	2.741 (1.400, 5.370)		2.690 (1.181, 6.129)	
	BWV, T3	3.180 (1.629, 6.209)		3.216 (1.384, 7.476)	
DM (-)	BWV, T1	Reference	0.877	Reference	0.459
	BWV, T2	1.316 (0.579, 2.995)		1.709 (0.527, 5.538)	
	BWV, T3	1.345 (0.591, 3.060)		1.786 (0.521, 6.128)	
DM (+)	BWV, T1	Reference		Reference	
	BWV, T2	1.990 (0.933, 4.246)		2.235 (0.868, 5.756)	
	BWV, T3	1.894 (0.898, 3.994)		2.398 (0.928, 6.194)	
eGFR ≥ 45 ml/min./1.73 m^2^	BWV, T1	Reference	0.743	Reference	0.970
	BWV, T2	1.742 (0.836, 3.631)		1.730 (0.687, 4.359)	
	BWV, T3	2.106 (1.036, 4.284)		2.513 (0.985, 6.411)	
eGFR <45 ml/min./1.73 m^2^	BWV, T1	Reference		Reference	
	BWV, T2	2.440 (1.134, 5.251)		3.071 (1.022, 9.225)	
	BWV, T3	2.237 (1.029, 4.859)		2.769 (0.895, 8.568)	
Spot urine ACR <300 mg/g	BWV, T1	Reference	0.081	Reference	0.151
	BWV, T2	1.011 (0.469, 2.180)		1.245 (0.461, 3.365)	
	BWV, T3	1.604 (0.791, 3.254)		2.461 (0.911, 6.652)	
Spot urine ACR ≥ 300 mg/g	BWV, T1	Reference		Reference	
	BWV, T2	3.672 (1.679, 8.031)		2.633 (0.928, 7.470)	
	BWV, T3	2.846 (1.289, 6.282)		2.606 (0.895, 7.589)	

*Models were adjusted for age, gender, Charlson comorbidity index, smoking history, BMI, SBP, DBP, medication (ACEi/ARBs, diuretics, number of antihypertensive drugs, statins), hemoglobin, albumin, HDL-C, fasting serum glucose, 25(OH) vitamin D, hs-CRP, eGFR, spot urine ACR, and baseline CACS*.

*ACR, albumin-to-creatinine ratio; BMI, body mass index; DM, diabetes mellitus; eGFR, estimated glomerular filtration rate, OR, odd ratio; T1, 1st tertile; T2, 2nd tertile; T3, 3rd tertile*.

## Discussion

In this study, we discovered that high BWV is independently associated with the rapid progression of CAC in patients with predialysis CKD. Importantly, the association between BWV and progression of CAC remains robust even among the subjects without significant BW gain or loss during follow-up periods.

Following a recent report that high BWV is associated with adverse CV outcomes in patients with predialysis CKD (18), we here suggest a rationale for the previous finding. Although BWV is associated with CV outcomes in patients with CAD (17), any direct evidence for the association between BWV and progression of CAC in patients with predialysis CKD has not been presented yet. On the other hand, compared to the patients with end-stage renal disease (ESRD), especially those under maintenance hemodialysis, the degree of BWV should be modest in patients with predialysis CKD. Intriguingly, the progression of CAC in patients with ESRD is altered by the modality of renal replacement therapy (30). A recent observational study reported that peritoneal dialysis is not associated with CAC or is associated with less CAC progression than hemodialysis (30). Provided that BWV is inevitable in most patients on hemodialysis, rather than in patients on peritoneal dialysis, primarily due to the discontinuous nature of hemodialysis leading to saw-tooth volume fluctuations (31), it is speculated that BWV may also be representative of ongoing CAC progression in patients with ESRD. Collectively, our finding expands the clinical significance of BWV as an indicator of underlying CAD in patients with predialysis CKD, which is in line with the findings both from the subjects without CKD and from the subjects with ESRD.

We are not able to present a precise mechanism of the association of BWV with the progression of CAC even in patients without significant longitudinal BW changes. One possible explanation is that BWV might be a mixed phenotype of the processes that facilitate BW gain and BW loss. As the accumulation of extracellular fluid and malnutrition-inflammation are not mutually exclusive, both of which is associated with CAC progression (1, 5, 6), a longitudinal trend of BW change may be masked if those processes concurrently take place. Therefore, a potential strength of BWV over longitudinal BW change may be a sensitive detection of vulnerable subjects who are at high risk of CAC progression, although the precise mechanism of the association of BWV with the progression of CAC should be further elucidated.

## Limitations

There are a number of limitations to this study. First, we were not able to determine the causal relationship between high BWV and progression of CAC, because of the observational nature of the current study. In addition, BWV was calculated from the data up to 8 years, while CACS was collected at the baseline and 4-year follow-up. Due to the limitation of the study design, it is also possible that CAC progression might promote BW fluctuation. A more focused study should evaluate the causal relation between BWV and the progression of CAC. Second, despite the clear association of high BWV with the progression of CAC, the precise mechanism should be further clarified. Third, although accumulation and removal of excess fluid have been suggested as a mechanism to explain BWV in the current study, the other cause, such as diet and physical activity, should also be considered, as BW was measured as long as every 12 months. Fourth, as this cohort study enrolled only ethnic Koreans, a precaution is required to extrapolate the data in this study to other populations.

## Conclusion

In conclusion, we report that high BWV is independently associated with the rapid progression of CAC in patients with predialysis CKD. Our results suggest that the association between BWV and progression of CAC remains robust even among the subjects without significant BW gain or loss during follow-up periods.

## Data Availability Statement

The raw data supporting the conclusions of this article will be made available by the authors, without undue reservation.

## Ethics Statement

The study was conducted in accordance with the principles of the Declaration of Helsinki, and the study protocol was approved by the institutional review boards of participating centers, including at Seoul National University Hospital, Yonsei University Severance Hospital, Kangbuk Samsung Medical Center, Seoul St. Mary's Hospital, Gil Hospital, Eulji General Hospital, Chonnam National University Hospital, and Pusan Paik Hospital. The patients/participants provided their written informed consent to participate in this study.

## Author Contributions

SS designed and helped in the data analysis and manuscript writing. SS, TO, and HC contributed to the conception of the study. SS and CK performed the data analyses and wrote the manuscript. EB, K-HO, K-BL, SH, and SS collected the data. SM and SK helped perform the analysis with constructive discussions. All authors contributed to the article and approved the submitted version.

## Funding

This study was supported by the Research Program funded by the Korea Centers for Disease Control and Prevention (2011E3300300, 2012E3301100, 2013E3301600, 2013E3301601, 2013E3301602, 2016E3300200, 2016E3300201, 2016E3300202, and 2019E320100) and by the National Research Foundation of Korea (NRF) funded by the Korea Government (MSIT) (NRF-2019R1A2C2086276).

## Conflict of Interest

The authors declare that the research was conducted in the absence of any commercial or financial relationships that could be construed as a potential conflict of interest.

## Publisher's Note

All claims expressed in this article are solely those of the authors and do not necessarily represent those of their affiliated organizations, or those of the publisher, the editors and the reviewers. Any product that may be evaluated in this article, or claim that may be made by its manufacturer, is not guaranteed or endorsed by the publisher.
