# Supplementary material for: Association of Body Weight Variability With Progression of Coronary Artery Calcification in Patients With Predialysis Chronic Kidney Disease
Source: Front Cardiovasc Med. 2022 Jan 26;8:794957. doi: 10.3389/fcvm.2021.794957 (PMC8826058; doi:10.3389/fcvm.2021.794957)
Supplement: Supplementary file 1 [file Data_Sheet_1.PDF]

Supplementary Material

Association of body weight variability with progression of coronary artery calcification in patients with pre-dialysis chronic kidney disease

Sang Heon Suh, M.D., Ph.D.[1], Tae Ryom Oh, M.D., Ph.D.[1], Hong Sang Choi, M.D., Ph.D.[1], Chang Seong Kim, M.D., Ph.D.[1], Eun Hui Bae, M.D., Ph.D.[1], Kook-Hwan Oh, M.D., Ph.D.[2], Kyu-Beck Lee, M.D., Ph.D.[3], Seung Hyeok Han, M.D., Ph.D.[4], Suah Sung, M.D., Ph.D.[5], Seong Kwon Ma*, M.D., Ph.D.[1], and Soo Wan Kim*, M.D., Ph.D.[1], on behalf of the Korean Cohort Study for Outcomes in Patients With Chronic Kidney Disease (KNOW-CKD) Investigators

[1]Department of Internal Medicine, Chonnam National University Medical School and Chonnam National University Hospital, Gwangju, Korea
[2]Department of Internal Medicine, Seoul National University Hospital, Seoul, Korea
[3]Department of Internal Medicine, Kangbuk Samsung Hospital, Sungkyunkwan University School of Medicine, Seoul, Republic of Korea
[4]Department of Internal Medicine, College of Medicine, Institute of Kidney Disease Research, Yonsei University, Seoul, Korea
[5]Department of Internal Medicine, Eulji Medical Center, Eulji University, Seoul, Korea

Running title: BW variability and CAC progression in CKD

Corresponding authors
*Seong Kwon Ma, M.D., Ph.D., Department of Internal Medicine, Chonnam National University Medical School, 42 Jebongro, Gwangju 61469, Korea, Tel: +82-62-220-6579, Fax: +82-62-225-8578, Email: drmsk@hanmail.net
*Soo Wan Kim, M.D., Ph.D., Department of Internal Medicine, Chonnam National University Medical School, 42 Jebongro, Gwangju 61469, Korea, Tel: +82-62-225-6271, Fax: +82-62-220-8578, Email: skimw@chonnam.ac.kr

Table of Contents

Figure S1. The proportion of the subjects with rapid progression of coronary artery calcification during 4-year follow-up period by BWV

Table S1. Baseline characteristics of study participants in the tertile by BWV

Table S2. Multivariate linear regression of BWV for CACS change during follow-up periods

Table S3. Binary logistic regression of BWV for rapid progression of CAC in subjects with CACS ≥ 10 AU at the baseline

Table S4. Binary logistic regression of BWV for rapid progression of CAC in subjects with eGFR ≥ 30 mL/min./1.73m^2 at the baseline

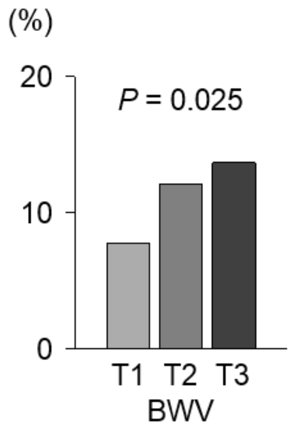

Figure S1. The proportion of the subjects with rapid progression of CAC during 4-year follow-up period by BWV.

Note: *P* value by Pearson's Chi square test. Abbreviations: BWV, body weight variability; T1, 1st tertile; T2, 2nd tertile; T3, 3rd tertile.

Table S1. Baseline characteristics of study participants in the tertile by BWV

	Longitudinal BW change			
	≤ -2.5%/year (n = 35)	-2.5 <, < 2.5%/year (n = 1087)	≥ -2.5%/year (n = 34)	*P* value
Follow-up duration (year)	5.762 ± 1.388	6.571 ± 1.401	5.872 ± 1.447	< 0.001
CACS (AU)				0.776
0	19 (54.3)	577 (53.1)	18 (52.9)	
0 <, ≤ 400	12 (34.3)	432 (39.7)	15 (44.1)	
400 <, ≤ 1000	2 (5.7)	53 (4.9)	1 (2.9)	
1000 <	2 (5.7)	25 (2.3)	0 (0.0)	
Age (year)	53.971 ± 14.443	52.713 ± 11.634	46.559 ± 13.734	0.009
Male	17 (48.6)	648 (59.6)	21 (61.8)	0.407
Charlson comorbidity index				0.181
0 – 3	26 (74.3)	899 (82.7)	27 (79.4)	
4 – 5	8 (22.9)	182 (16.7)	6 (17.6)	
6 – 7	1 (2.9)	6 (0.6)	1 (2.9)	
DM	278 (25.6)	12 (34.3)	8 (23.5)	
CAD	2 (5.7)	19 (1.7)	0 (0.0)	
Arrhythmia	0 (0.0)	15 (1.4)	0 (0.0)	
Medication				
ACEi/ARBs	33 (64.3)	941 (86.6)	28 (82.4)	0.314
Diuretics	10 (28.6)	266 (24.5)	14 (41.2)	0.077
Number of anti-HTN drugs ≥ 3	10 (28.6)	252 (23.2)	8 (23.5)	0.759
Statins	20 (57.1)	548 (50.4)	12 (35.3)	0.156
BMI (kg/m^2)	24.251 ± 3.868	24.535 ± 3.298	23.747 ± 3.525	< 0.001
SBP (mmHg)	126.257 ± 13.241	126.009 ± 14.590	123.235 ± 17.960	0.550
DBP (mmHg)	76.286 ± 9.963	76.916 ± 10.336	76.647 ± 12.373	0.931
Laboratory findings				
Hemoglobin (g/dL)	12.988 ± 1.946	13.374 ± 1.808	13.164 ± 2.383	0.413
Albumin (g/dL)	4.126 ± 0.341	4.272 ± 0.339	4.165 ± 0.488	0.012
Total cholesterol (mg/dL)	177.882 ± 35.476	174.468 ± 35.511	183.882 ± 44.476	0.282
HDL-C (mg/dL)	51.082 ± 14.746	50.843 ± 14.907	49.063 ± 18.048	0.798

LDL-C (mg/dL)	102.212 ± 31.572	96.559 ± 29.775	106.859 ± 35.276	0.086
TG (mg/dL)	146.882 ± 79.444	152.868 ± 96.326	150.636 ± 77.853	0.930
Fasting glucose (mg/dL)	109.471 ± 27.617	106.777 ± 30.930	99.647 ± 15.111	0.350
25(OH) vitamin D	16.577 ± 7.976	18.379 ± 7.243	16.736 ± 7.855	0.172
hsCRP (mg/dL)	0.600 [0.200, 1.900]	0.600 [0.100, 1.600]	0.490 [0.200, 1.350]	0.929
Spot urine ACR (mg/gCr)	400.951 [93.797, 1049.202]	226.894 [43.171, 589.640]	345.961 [89.139, 976.010]	0.156
eGFR (mL/min./1.73m^2)	53.485 ± 31.379	59.434 ± 28.385	63.180 ± 39.492	0.358
CKD stages				0.043
Stage 1	6 (17.1)	232 (21.3)	11 (32.4)	
Stage 2	9 (25.7)	280 (25.8)	5 (14.7)	
Stage 3a	3 (8.6)	216 (19.9)	4 (11.8)	
Stage 3b	8 (22.9)	242 (22.3)	7 (20.6)	
Stage 4	8 (22.9)	111 (10.2)	6 (17.6)	
Stage 5	1 (2.9)	6 (0.6)	1 (2.9)	

Note: Values for categorical variables are given as number (percentage); values for continuous variables, as mean ± standard deviation or median [interquartile range]. Abbreviations: ACEi, angiotensin-converting enzyme inhibitor; ACR, albumin-to-creatinine ratio; ARB, angiotensin receptor blocker; AU, Agatston unit; BMI, body mass index; BW, body weight; CACS, coronary artery calcium score; CAD, coronary artery disease; CKD, chronic kidney disease; Cr, creatinine; DBP, diastolic blood pressure; DM, diabetes mellitus; eGFR, estimated glomerular filtration rate; HDL-C, high density lipoprotein cholesterol; hsCRP, high-sensitivity C-reactive protein; HTN, hypertension; LDL-C, low density lipoprotein cholesterol; SBP, systolic blood pressure; T1, 1st tertile; T2, 2nd tertile; T3, 3rd tertile; TG, triglyceride; 25(OH) vitamin D, 25-hydroxyvitamin D.

Table S2. Multivariate linear regression of BWV for CACS change during follow-up periods

	Unadjusted		Adjusted	
	β coefficient (95%CIs)	*P* for interaction	β coefficient (95%CIs)	*P* for interaction
All subjects	11.536 (-1.145, 24.217)		12.098 (1.807, 22.389)	
Age < 60 years	8.496 (-3.376, 20.367)	0.051	5.965 (-3.478, 15.408)	0.265
Age ≥ 60 years	30.422 (-1.749. 62.593)		24.620 (-2.497, 51.738)	
Male	6.155 (-11.661, 23.971)	0.244	11.105 (-3.392, 25.601)	0.939
Female	14.758 (-0.800, 30.316)		10.739 (-2.130, 23.608)	
Charlson comorbidity index ≤ 3	2.248 (-6.748, 11.244)	0.025	5.734 (-1.954, 13.422)	0.04
Charlson comorbidity index ≥ 4	35.169 (-21.618, 91.957)		37.234 (13.790, 88.258)	
DM (-)	-0.913 (-8.669, 6.842)	0.04	2.127 (-4.258, 8.511)	0.153
DM (+)	22.761 (-17.061, 62.583)		27.669 (-6.579, 61.917)	
eGFR ≥ 45 mL/min./1.73m^2	14.268 (0.355, 28.182)	0.960	13.179 (1.556, 24.803)	0.190
eGFR < 45 mL/min./1.73m^2	7.135 (-20.369, 24.638)		5.819 (-16.580, 28.217)	
Spot urine ACR < 300 mg/g	6.509 (-7.762, 20.781)	0.536	10.300 (-1.262, 21.861)	0.991
Spot urine ACR ≥ 300 mg/g	14.039 (-7.794, 35.872)		14.62 (-3.251, 32.494)	

Note: Models were adjusted for age, gender, Charlson comorbidity index, smoking history, BMI, SBP, DBP, Medication (ACEi/ARBs, diuretics, number of antihypertensive drugs, statins), hemoglobin, albumin, HDL-C, fasting serum glucose, 25(OH) vitamin D, hs-CRP, eGFR, spot urine ACR, and baseline CACS. Abbreviations: BWV, Body weight variability; CI, confidence interval; OR, odd ratio; T1, 1st tertile; T2, 2nd tertile; T3, 3rd tertile.

Table S3. Binary logistic regression of BWV for rapid progression of CAC in subjects with CACS ≥ 10 AU at the baseline

	Unadjusted		Adjusted	
	OR (95%CIs)	*P* value	OR (95%CIs)	*P* value
BWV, T1	Reference		Reference	
BWV, T2	2.051 (1.142, 3.683)	0.016	2.331 (1.151, 4.718)	0.019
BWV, T3	2.134 (1.183, 3.848)	0.012	2.380 (1.148, 4.932)	0.020

Note: Models were adjusted for age, gender, Charlson comorbidity index, smoking history, BMI, SBP, DBP, Medication (ACEi/ARBs, diuretics, number of antihypertensive drugs, statins), hemoglobin, albumin, HDL-C, fasting serum glucose, 25(OH) vitamin D, hs-CRP, eGFR, spot urine ACR, and baseline CACS. Abbreviations: BWV, Body weight variability; CI, confidence interval; OR, odd ratio; T1, 1st tertile; T2, 2nd tertile; T3, 3rd tertile.

Table S4. Binary logistic regression of BWV for rapid progression of CAC in subjects with eGFR ≥ 30 mL/min./1.73m^2 at the baseline

	Unadjusted		Adjusted	
	OR (95%CIs)	*P* value	OR (95%CIs)	*P* value
BWV, T1	Reference		Reference	
BWV, T2	2.271 (1.271, 4.059)	0.006	2.291 (1.078, 4.867)	0.031
BWV, T3	2.393 (1.344, 4.261)	0.003	3.328 (1.529, 7.242)	0.002

Note: Models were adjusted for age, gender, Charlson comorbidity index, smoking history, BMI, SBP, DBP, Medication (ACEi/ARBs, diuretics, number of antihypertensive drugs, statins), hemoglobin, albumin, HDL-C, fasting serum glucose, 25(OH) vitamin D, hs-CRP, eGFR, spot urine ACR, and baseline CACS. Abbreviations: BWV, Body weight variability; CI, confidence interval; OR, odd ratio; T1, 1st tertile; T2, 2nd tertile; T3, 3rd tertile.
